# Assessing the Perceptions of Inspiratory Muscle Training in Children With Cystic Fibrosis and Their Multidisciplinary Team: Mixed-Methods Study

**DOI:** 10.2196/11189

**Published:** 2018-10-25

**Authors:** Jessica L McCreery, Kelly A Mackintosh, Narelle S Cox, Melitta A McNarry

**Affiliations:** 1 School of Sport and Exercise Sciences College of Engineering Swansea University Swansea United Kingdom; 2 School of Allied Health Department of Rehabilitation, Nutrition and Sport La Trobe University Melbourne Australia

**Keywords:** cystic fibrosis, health perceptions, inspiratory muscle training, mobile phone, pediatrics, qualitative

## Abstract

**Background:**

Little is known about the opinions or perceived benefits of an inspiratory muscle training intervention in patients with cystic fibrosis and their multidisciplinary team.

**Objective:**

The aim of this qualitative study was to examine patients' and multidisciplinary teams' views on inspiratory muscle training to inform and tailor future interventions.

**Methods:**

Individual, semistructured interviews were conducted to evaluate participants’ perspectives of a 4-week inspiratory muscle training intervention. In this study, 8 of 13 individuals involved in the inspiratory muscle training program (5 children aged 11-14 years; 2 physiotherapists; and 1 respiratory physician) participated. Interviews were transcribed verbatim, analyzed using thematic analyses, and then coded into relevant themes.

**Results:**

Four key themes emerged: acceptability, facilitators, barriers, and recommendations. While fun, enjoyment, and improved perceived physical ability were reported by children and their multidisciplinary team following the inspiratory muscle training program, the multidisciplinary team identified factors such as time and cost as key barriers.

**Conclusions:**

A short inspiratory muscle training program was perceived to have positive effects on the physical ability and psychosocial health of children with cystic fibrosis. These findings highlight the importance of obtaining participants’ and multidisciplinary teams' perceptions and recommendations to ensure the efficacy and optimal design of future inspiratory muscle training protocols.

## Introduction

Cystic fibrosis (CF) is the most common inherited, life-shortening condition in the United Kingdom [1]. Despite recent advances in pharmacological interventions [2], the median life expectancy remains around 40 years [1]. Characterized by recurrent respiratory infections, breathlessness, cough, and gastrointestinal complications, CF is a multisystem disease requiring many hours of daily therapy [3]. With no cure available currently, the development or refinement of treatment strategies that increase or maintain the quality of life (QoL), exercise capacity, and respiratory function are paramount for the well-being of patients with CF.

Inspiratory muscle training (IMT), which utilizes restricted airflow breathing exercises to increase the mechanical load on the external intercostal muscles and diaphragm, is a subject of research interest. The increased muscular load engendered by IMT provides a stimulus to elicit a hypertrophic response [4], similar to that observed in response to strength training in limb muscles [5]. Substantial improvements in respiratory muscle endurance [6,7], respiratory muscle strength, and vital capacity [8] have been reported in patients with CF who have undertaken IMT, and there is some evidence for a positive effect on lung function and QoL [4]. Despite the potential benefits of IMT, there is a lack of consensus regarding its routine use in clinical practice due to marked variations in study protocols, small sample sizes, and lack of psychosocial outcome measures [5,9]. Specifically, only two studies have reported psychosocial health as an outcome, reporting that anxiety and depression scores decreased in an IMT group that trained at 80% of their maximal effort [4] and a trend toward an improved QoL with a combined IMT “whole muscle” training program [10]. Regardless of potential efficacy, many treatment strategies are limited by participants’ perceptions of, and, thus, adherence to, the intervention. Indeed, a common barrier to adherence cited by many adolescents with CF is the embarrassment of taking their treatments in front of other people [11,12]; this suggests the potential utility of home-based interventions, such as IMT, which may increase adherence to treatments in adolescents with CF. However, no studies are presently available regarding the perceptions, opinions, or recommendations of participants, or indeed of a multidisciplinary team (MDT), concerning IMT. Furthermore, the mean age of participants in IMT studies is approximately 18.5 years [13]. With the average life expectancy of around 45.1 years, participants in these studies are effectively middle aged, and therefore, further research in the younger CF population is warranted [14]. This lack of evidence makes it difficult to establish the overall efficacy of IMT as a therapeutic strategy for adolescents with CF [9]. Therefore, the aim of this study was to ascertain the views of children, and their respective CF MDT, in relation to IMT following a 4-week training program.

## Methods

In this study, 5 children (age, 11-14 years) were included if they met the following criteria: (1) took part in the pilot study of IMT conducted by Swansea University; (2) had a confirmed diagnosis of CF or were a matched control; (3) had no additional non-CF illness or disease; and (4) voluntarily participated and provided written informed parental consent and child assent. MDT participants (2 physiotherapists and 1 respiratory physician) were eligible for inclusion if they provided clinical care for children with CF and had been involved in the same IMT pilot study.

### Inspiratory Muscle Training

The IMT intervention consisted of participants undertaking 30 inspirations, twice a day, for 28 consecutive days using a POWERbreathe Plus device (POWERbreathe Plus LF Level 1, Gaiam Ltd E & OE, UK). A progressive approach was adopted, whereby participants initially trained at a load of 40% of their baseline maximal inspiratory pressure, increasing to 50% during weeks 3 and 4 [10]. All procedures and protocols utilized in this study were approved by the local National Health Service (NHS) ethical committee (13/LO/1907).

### Qualitative Protocol—Interviews

Children and MDT members took part in individual semistructured interviews with follow-up questions. A semistructured interview includes a series of predetermined but open-ended questions, thereby allowing the interviewer to follow topical trajectories in the conversation, as well as providing interviewees freedom to express their views in their own terms [15,16]. Children’s interview questions were related to their thoughts and opinions of IMT. MDT’s interview questions were centered around their opinions of IMT, responses from patients, and IMT reflections and recommendations. All interviews were conducted by one investigator (JLM). Table 1 presents sample interview questions. All interviews were recorded and transcribed verbatim for analysis.

**Table 1:** Example interview questions.

Interview and topic		Examples
**Children**		
	Inspiratory muscle training	What did you like and dislike about the training device? What made it easy or difficult to do the training program?
	Future	How would you react if you were asked to do this training again?
**Multidisciplinary team**		
	Inspiratory muscle training	What is your opinion of the IMT device? How did the patients respond to the IMT device?
	Future	What do you think about the National Health Service adopting an IMT intervention as a treatment? What recommendations do you have for interventions you would like to see for CF patients?

### Data Analysis

All interviews were transcribed verbatim by one author (JLM) and analyzed thematically [17] using a manual approach. One author (JLM) read and familiarized the transcripts, and an initial list of codes was developed to organize the data to identify and develop themes from them [18]. A cross-examination of thematic data was undertaken by the research team in reverse, tracing verbatim quotations back to transcripts to ensure that the developed themes were grounded in the original data [19]. To ensure methodological rigor, themes and verbatim quotations were then reviewed by 3 authors to ensure findings were worthy of attention and to offer an alternative interpretation of the data [20,21]. This process continued until an acceptable consensus had been reached by the group.

## Results

Five children (n=3 boys) and 3 MDTs (2 physiotherapists and 1 respiratory physician) completed the interviews. All interviews were semistructured and lasted between 30 and 40 minutes. Example verbatim quotes are provided to support the points raised with a frequency count (in brackets) to indicate the number of times the particular theme was raised. The following 4 themes emerged from the interviews: (1) acceptability; (2) facilitators; (3) barrier; and (4) recommendations.

### Acceptability

Feedback from all participants was very positive regarding the acceptability of the intervention. The MDT noted children’s enthusiasm, and all children reported enjoying the IMT intervention:

I felt really excited [to do IMT] I just wanted to have a go at it.

Girl; n=5

Some patients even suggested they would like to take part in future interventions:

I was pretty sad I didn’t have to do it again. I’d happily do it again.

Girl; n=4

Children reported good participation and adherence to the IMT intervention due to the ease of implementation:

Like it was easy you just do it [IMT] at home. You don’t have to go anywhere specific or special. You could just do it in your bedroom if you wanted.

Girl; n=2

In addition, the ease of integration into daily routine:

I got into it…I feel like I’ve always done it. When you get up in the morning, you normally eat your breakfast, get ready for school, do IMT and then quickly leave the house for the bus.

Girl; n=5

Most importantly, children expressed enthusiasm and enjoyment:

I really enjoyed it, I got into it…I feel like I’ve always done it.

Boy; n=4

Furthermore, all children perceived IMT to have a positive effect on their ability when partaking in a physical activity (PA):

For some reason, I don’t know how, but they [lungs] almost felt like, almost got stronger. You could just breathe more freely…I could keep running for longer, I didn’t have to stop and take deep breaths as much as normal.

Girl; n=5

Children also indicated reduced embarrassment associated with completing the treatment at home:

I like it [using the device at home] cause of not having all the constant questions. I don’t like, well, I don’t mind answering questions when people do ask [about CF], but like I am not getting caught up in it all the time in school with my friends.

Boy; n=2

Moreover, the CF care team reported positive feedback from their patients and high adherence:

They would have all liked to have kept the IMT device and carried on. In fact, one of the patients subsequently went out and bought one and uses it as part of their routine.

Female Physiotherapist; n=3

Akin to the children’s perceptions, CF care team members believe that the IMT training schedule fit well around children’s home and school schedules:

From a practical point of view, I think that fitted well. All feedback seemed to confirm that.

Female Physiotherapist; n=2

### Facilitators

Unsurprisingly, the MDT highlighted the importance of family, specifically parental influence, with “sporty families” being labeled as easier to motivate to undertake an intervention and exercise:

If you’ve a sporty family its easier…Families will support them [children] most of the families were very keen on IMT. You have to get the families on board.”

Female Physiotherapist; n=3

Not only are family facilitators influential, but peer facilitators are key, especially for children. The MDT highlighting the importance of children with CF being seen as equal to their peers:

It is very important to both keep them healthy and also to keep them in their peer group, you know at school and during sports activities. They need to be able to keep up with the rest of their class, so it is very, very important…A psychological benefit of being able to keep up with their peer group.

Female Physiotherapist; n=3

### Barriers

Although it seemed the intervention was well adhered to and enjoyed thoroughly by all participants, barriers were nonetheless highlighted, although predominantly by the MDT rather than the children. The MDT highlighted the following main barriers to the implementation of IMT:

#### Cost

The clinical care team highlighted cost to be a major barrier in implementing IMT within the local NHS framework:

Obviously there is a cost implication as there is no money in the NHS for any of these things.

Female Respiratory Physician; n=3

Furthermore, there is a reliance on charitable income to fund airway clearance equipment:

Cost would be a big thing and whether it was a benefit to patients.

Female Respiratory Physician; n=3

However, it was accentuated that if IMT proved to be successful, the cost should be met:

If it is proven to be beneficial they [the NHS] are more likely to get it. If it’s proven to be beneficial and improve lung function compared to the cost of some of the drugs they [the NHS] might pay for it.

Female Physiotherapist; n=3

#### Burden

Patients with CF have a high treatment burden involving daily physical therapy coupled with medication. Incorporating IMT into an already busy treatment schedule was a concern raised by the MDT:

It’s yet another thing for us to ask them to do because they do have quite a large treatment burden…so that would be the biggest con, a time thing.

Female Physiotherapist; n=3

This concern was also echoed by one of the children:

It was extra work to do with everything else that I have to do.

Boy; n=2

Yet, one of the MDT had a solution, whereby an IMT program could be viable within the treatment schedule of a patient with CF:

Obviously you don’t want it to be too much of a burden. Deciding whether it is better than other parts of their treatment and other part of their Physio and then substitute it [IMT] in could be an option.

Female Respiratory Physician; n=1

#### Family

Converse to families being deemed as facilitators, a physiotherapist reflected on previous cases, whereby children’s divorced parents have had a negative impact on children’s participation levels in activities:

The parents have divorced, and the girl lives with mum. The mum has a full-time job and mum didn’t push any after-school clubs, dad was the one that did it previously. So that created a big barrier.

Female Physiotherapist; n=1

### Recommendations

Participants were requested to comment on any changes they would make to the device and protocol. The children reported no changes, whereas the care team had numerous suggestions to improve future interventions. One of the main changes the care team suggested was the importance of knowing whether participants were adhering to the intervention:

I don’t know if you can measure compliance, but it would be good if it [IMT] can tell us how much they actually did.

Female Respiratory Physician; n=1

To ensure future would adherence to IMT protocols and make the intervention attractive to children, the following was suggested:

Young people like to have their smartphones and apps, visual feedback in a piece of electrical equipment, that is probably the way forwards.

Female Respiratory Physician; n=2

In addition, implementing a competitive element was highlighted as important additions in future interventions:

Feedback so they know how well they are doing. They are quite competitive, so if they know the others are doing it, they’ll be more motivated.

Female Physiotherapist; n=2

Contrastingly, despite reporting that a three times-a-day intervention fitted well into a child’s routine, the MDT suggested a more time-efficient intervention to reduce the burden on patients:

Three times a day would be a problem, it [IMT] would have to be something regular to get them into a routine.

Female Respiratory Physician; n=3

## Discussion

The aim of this study was to ascertain the views of children and the CF care team in relation to an IMT intervention, thereby providing population-specific evidence to inform future interventions. Results indicate that all children enjoyed the home-based intervention, while the CF care team raised concerns regarding cost and treatment burden. Overall, these results provide important insights regarding future IMT interventions, building upon the limited literature available regarding the opinions of patients, respiratory physicians, and physiotherapists.

IMT in patients with CF has been reported to improve endurance and strength of the inspiratory muscles, as well as exercise capacity [5]. Previous research has shown that increases in exercise capacity are associated with the improved psychosocial status in patients with chronic pulmonary disease [22]. In addition, a recent study found that aerobic fitness positively associated with health-related QoL in patients with CF, underlining the importance of good physical fitness [23]. However, whether greater perceived ability to be physically active has the potential to influence psychosocial health and QoL in patients with CF remains unknown.

The perceived improvement in physical ability reported by participants could be attributed to the good adherence to the IMT program. This is in contrast to previous reports that adherence to treatment in CF is suboptimal [24]. This discrepancy could be a result of our participants’ MDT, the age of our participants, and the small numbers involved in the study. Adherence levels among patients with CF tend to decline with the increasing age [25]. In younger children, treatment responsibility often lies with parents or guardians, resulting in greater adherence. As adherence was self-reported by participants in this study, it may have been over- or underestimated and is, subsequently, subject to the risk of bias [26]. Key factors that influence adherence include family environment, stigma, embarrassment among peers, and relationship with their MDT [24]. Indeed, the interview findings presented here reflect an MDT that actively encourage patients to make their own choices about treatment decisions and are open to trialing new or novel interventions such as IMT.

With a reduced exercise capacity and low daily PA levels potentially impacting psychological and physiological health of patients with CF, parental and family involvement in PA is extremely important when encouraging children to meet recommended PA and exercise guidelines [27], which can be translated to IMT interventions. Healthy children with physically active parents are over five times more likely to be active than children whose parents are inactive [28], which correlates with reports from the MDT that an active “sporty family” is essential regarding children’s participation levels. In addition, children’s moderate-to-vigorous PA (MVPA) levels have been shown to correlate with their parents’ [29], with family cohesion and parent-child joint PA predicting higher levels of MVPA [30]. Conversely, divorced parents can have a negative impact on a child’s PA levels when the main parental facilitator becomes less involved [31]; this impact of divorced parents was highlighted by the MDT.

In addition to families, peer support is influential in determining activity-related self-esteem and, therefore, treatment behaviors[32]. School-aged children with CF report concerns of appearing “different” than their peers [33], and our participants voiced that they like to keep their condition separate from their friendships at school. This is in accordance with research in which children and adolescents with CF attempted to conceal their disease and symptoms to appear “normal” to their peers [34]. As the perspectives of peers are critical to social acceptance, it is essential that children with CF are not defined by their disease but have their own identity. The time-efficient nature of IMT provides the capacity for self-directed therapy that does not detract from time spent with peers, which may increase adherence.

Despite the ease of implementation, good adherence, and enjoyment reported by participants, the MDT was more reserved with regards to their enthusiasm for an IMT intervention, with reservations relating to cost and treatment burden. Nevertheless, the team expressed an interest in investigating the potential of a longer-term IMT intervention to provide a clearer evidence-base on the impact on psychological and physiological health in patients with CF. The main concern voiced by the care team, as well as children with CF, was the potential burden it may have on patients in terms of their time and current treatments. Reports that a patient with CF can spend a mean of 108 minutes per day on a wide range of CF therapies, regardless of age or disease severity [3], highlights the importance of establishing the feasibility of time-efficient therapies such as IMT. In addition, the CF care team recommended that future IMT interventions incorporate technology, including the ability to monitor adherence objectively to reduce the risk of bias, linking to smartphones and providing visual feedback. With smartphone ownership increasing, its usability for future interventions is highlighted by its accessibility, real-time assessment, adherence monitoring, visual feedback, and adjustability to the user [35]. Increasing patient and MDT engagement in the intervention design has the potential to improve health outcomes, better patient care, and lower costs [36] and, furthermore, is essential in improving the quality of health care and efficacy, which is especially important for the CF population whose treatments are highly prescribed [37].

In conclusion, the data revealed consistent themes relating to IMT among children with CF and their MDT. This preliminary study highlights the ease of incorporating an IMT program into the lives of patients with CF, who reported noticeable perceived improvements to their physical ability after only 4 weeks of IMT. These preliminary results suggest that an IMT intervention may be well accepted by young patients with CF. Furthermore, this study emphasizes the importance of gathering views and opinions of patients and their care teams to ensure good adherence and enjoyment to future interventions.

## Abbreviations

CF: cystic fibrosis
IMT: inspiratory muscle training
MDT: multidisciplinary team
MVPA: moderate-to-vigorous physical activity
NHS: National Health Service
PA: physical activity
QoL: quality of life
